# Multifaceted anti-colorectal tumor effect of digoxin on HCT8 and SW620 cells *in vitro*

**DOI:** 10.1093/gastro/goaa076

**Published:** 2020-12-10

**Authors:** Yong-Qiang Hou, Ying-Ying Wang, Xing-Can Wang, Yao Liu, Chun-Ze Zhang, Zhe-Sheng Chen, Zhe Zhang, Wei Wang, De-Xin Kong

**Affiliations:** 1 Tianjin Key Laboratory on Technologies Enabling Development of Clinical Therapeutics and Diagnostics, School of Pharmaceutical Sciences, Tianjin Medical University, Tianjin, P. R. China; 2 Department of Otorhinolaryngology Head and Neck, Institute of Otorhinolaryngology, Tianjin First Central Hospital, Tianjin, P. R. China; 3 Department of Colorectal Surgery, Tianjin Union Medical Center, Tianjin, P. R. China; 4 Department of Pharmaceutical Sciences, College of Pharmacy and Health Sciences, St. John's University, Queens, NY, USA; 5 School of Medicine, Tianjin Tianshi College, Tianyuan University, Tianjin, P. R. China

**Keywords:** digoxin, antitumor, colorectal cancer, cell-cycle arrest, metastasis, multidrug resistance

## Abstract

**Background:**

Colorectal cancer (CRC) is one of the leading causes of cancer death worldwide. Novel drugs for CRC therapy are urgently needed. Digoxin has been in clinical use for treatment of heart failure and atrial arrhythmias for many years. Fragmentary reports suggested that digoxin might have antitumor efficacy on CRC. Here, we aimed to investigate the antitumor effect of digoxin on human CRC cells and the underlying mechanism.

**Methods:**

Cell viability was determined using 3-(4,5-dimethyl-2-thiazolyl)-2,5-diphenyl-2-H-tetrazolium bromide (MTT) assay and plate colony formation assay. The effects of digoxin on cell-cycle distribution and apoptosis were analysed by flow cytometry. The anti-metastatic effect on tumor cells was determined by wound-healing assay and transwell assay. Anti-angiogenic effect was examined by determining the inhibition against proliferation, migration, and tube formation of human umbilical vein endothelial cells (HUVECs). Mechanism study was performed by Western blot, enzyme-linked immunosorbent assay (ELISA), and gelatin-zymography assay.

**Results:**

Digoxin potently inhibited cell proliferation, induced G1-phase and G2/M-phase arrest in colorectal-cancer HCT8 and SW620 cells, respectively. No obvious apoptosis was observed in the treated cells. Anti-metastatic activities were shown on HCT8 cells by inhibiting the migration and invasion. Meanwhile, the expression of MMP2, MMP9, and phosphorylated Integrinβ1 were decreased. Digoxin inhibited the proliferation, migration, and tube formation of HUVECs and reduced HIF1α expression and vascular endothelial growth factor A (VEGF-A) secretion in HCT8 cells, suggesting anti-angiogenic activity. Furthermore, digoxin significantly reversed ABCB1-mediated multidrug resistance on SW620/Ad300 cells.

**Conclusion:**

Our findings suggest that digoxin has the potential to be applied as an antitumor drug via inhibiting proliferation and metastasis as well as reversing the ABCB1-mediated multidrug resistance of colorectal cancer.

## Introduction

Colorectal cancer (CRC) is one of the most common malignant tumors worldwide. Although numerous exciting advances in the treatment of CRC have been made over the past decades, metastasis and multidrug resistance remain the major causes of CRC-related mortality [1, 2]. Metastasis is a multi-step process involving the dissemination of cancer cells to distant metastatic sites, travel in the blood and lymphatic circulation, and the formation of metastatic colonies in distant tissues, ultimately causing the death of cancer patients [3]. Multidrug resistance is a critical factor responsible for the failure of chemotherapeutics, also leading to the poor survival rate of patients with CRC [4].

Chemotherapeutics remains an important approach for treating metastatic CRC (mCRC). The combinations of cytotoxic agents such as fluorouracil, leucovorin, and irinotecan are the first-line treatments [5]. Targeting tumor angiogenesis is another therapy for mCRC. The vascular endothelial growth factor A (VEGF-A)-targeting monoclonal antibody, bevacizumab, is the first anti-angiogenic agent approved by the US Food and Drug Administration for mCRC treatment [6]. On the other hand, multidrug resistance is known to be a major obstacle for chemotherapy of patients with CRC [7]. The ATP-binding cassette (ABC) transporters have been reported to be upregulated in colorectal cancer, facilitating the efflux of anticancer drugs and therefore decreasing intracellular drug accumulation [8]. Thus, a higher dosage of chemotherapy drugs was needed, causing unnecessary toxicities. Above all, novel drugs for the therapy of CRC with metastasis and multidrug resistance remain urgently needed.

Digoxin has been widely used for the treatment of heart failure and atrial arrhythmias for many years. Accumulating evidence has suggested that it might be a candidate for cancer treatment. Early studies reported that the tumor volume of patients with breast cancer reduced after treatment with digoxin [9–11]. Moreover, the recurrence rate of patients with breast cancer who received digoxin was significantly lower than for those without digoxin treatment [12]. Recently, plenty of clinical trials with digoxin as an anticancer drug candidate, alone or in combination with chemotherapeutic drugs were reported [13–15]. For CRC patients, digoxin was proved to be safe [16]. However, the detailed antitumor mechanisms, particularly whether digoxin could inhibit cancer metastasis as well as reverse drug resistance in colorectal cancer, remain unknown.

Recently, we investigated the *in vitro* antitumor effect including the anti-metastatic effect and multidrug resistance-reversing effect of digoxin on CRC by using HCT8, SW620, and SW620/Ad300 cells.

## Materials and methods

### Reagents

Digoxin was purchased from Aladdin (London, Ontario, Canada). Doxorubicin was obtained from Dalian Meilun Biological Product Factory (Dalian, Liaoning, China). Cisplatin and verapamil were purchased from Energy Chemical (Shanghai, China). Anti-CyclinD1, anti-Cdc2, anti-CyclinB1, anti-HIF1α, anti-p-Rb (phospho S780), and anti-β-actin antibodies, as well as anti-mouse and anti-rabbit horseradish peroxidase (HRP)-conjugated secondary antibodies, were obtained from Cell Signaling Technology (Danvers, MA, USA). Anti-p21 was purchased from Santa Cruz Biotechnology (Santa Cruz, CA, USA). Anti-p-Integrinβ1 (phospho T788+T789) was from Abcam (Cambridge, MA, USA). Anti-MMP2 and anti-MMP9 were obtained from Bioss (Beijing, China). Matrigel and Annexin V-fluorescein isothiocyanate/propidium iodide (Annexin V-FITC/PI) apoptosis-detection kits were from BD Biosciences (San Jose, CA, USA). Propidium iodide (PI) was from Sigma-Aldrich (St. Louis, MO, USA). Human VEGF-A kit was purchased from Jianglai biotech (Shanghai, China). 3-(4,5-dimethyl-2-thiazolyl)-2,5-diphenyl-2-H-tetrazolium bromide (MTT) reagent was from Amresco (Solon, OH, USA).

### Cell culture

Human CRC cells HCT8 and SW620 were obtained from Cell Resource Center, Peking Union Medical College (Beijing, China). The SW620 cell line and its doxorubicin-selected ABCB1-overexpressing SW620/Ad300 cell line were a gift from Drs. Susan E. Bates and Robert W. Robey (National Cancer Institute (NCI), National Institutes of Health (NIH); Bethesda, MD, USA) and they were used for the ABCB1 reversal study. Human umbilical vein endothelial cells (HUVECs) were purchased from Cell Bank of Chinese Academy of Sciences (Shanghai, China). HCT8 and SW620 cells were cultured in Roswell Park Memorial Institute (RPMI) 1640 medium supplemented with 10% fetal bovine serum (FBS) and SW620/Ad300 cells were maintained in medium with 300 ng/mL doxorubicin. HUVECs were cultured in Dulbecco's Modified Eagle's Medium supplemented with 10% FBS. All the cells were cultured at 37°C in a humidified atmosphere containing 5% CO_2_. Drug-resistant cells were grown in drug-free culture media for >2 weeks before assay.

### Cell viability and multidrug-resistance-reversal assay

Cell viability and multidrug-resistance-reversal fold were determined using MTT assay. Briefly, HCT8 and SW620 cells were separately seeded into 96-well plates at a density of 4 × 10^4^ cells/mL (200 μL per well). Each cell line was treated with various concentrations of digoxin for 24 h. Two hundred microliters of HUVECs (4 × 10^4^ cells/mL) were cultured in a 96-well plate with supernatant of HCT8 cells pretreated with digoxin for 24 h. To determinate the reversal fold values, SW620 and SW620/Ad300 cells were separately cultured in 96-well plates at a density of 1 × 10^4^ cells/mL (200 μL per well). The SW620 and SW620/Ad300 cells were treated with digoxin and verapamil for 2 h, respectively. Then, these four groups were separately treated with doxorubicin or cisplatin and co-incubated for 72 h. Finally, MTT solution (5 mg/mL) was added to each well and the cells were further incubated for 4 h. The produced formazan blue was dissolved with dimethyl sulfoxide (DMSO) and the absorbance was measured at 490 nm using a microplate reader (Bio-Rad; Hercules, CA, USA).

### Plate colony formation assay

Plate colony formation assay was used to determine the tumorigenicity of HCT8 and SW620 cells. Cells were seeded into six-well plates at a density of 200 cells per well and cultured with RPMI 1640 medium containing 10% FBS. After 24 h of treatment with the indicated concentrations of digoxin, the culture medium was replaced every 3 days. After culture for 12 days at 37°C with 5% CO_2_, the colonies were fixed with 4% paraformaldehyde followed by 30 min of crystal violet (0.5%) staining. Colonies >0.1 mm in diameter were counted using Image J software (NIH; Bethesda, MD, USA).

### Flow cytometry for cell-cycle-distribution analysis

Cell-cycle distribution was analysed by PI labeling after the cells were treated with digoxin. HCT8 and SW620 cells were seeded into six-well plates (2 × 10^5^ cells/well) and treated with different concentrations of digoxin for 24 h. The cells were fixed in ice-cold ethanol (70%) for 30 min and suspended in phosphate buffered saline (PBS) containing 0.1% Triton X-100. After that, the cells were incubated in 50 μg/mL of PI solution for 30 min and then analysed by flow cytometer FACS Verse (BD Biosciences). Data were analysed using Flow Jo Software (Tristar; Long Beach, CA, USA).

### Flow cytometry for cell-apoptosis analysis

Annexin V/PI staining assay was employed to evaluate the apoptotic rate. HCT8 and SW620 cells were seeded into six-well plates (2 × 10^5^ cells/well) and treated with different concentrations of digoxin for 24 h. The treated cells were then collected and incubated with Annexin V-FITC and PI in the dark for 20 min. Finally, the cells were resuspended in binding buffer and detected using a flow cytometer FACS Verse (BD Biosciences). Data were analysed using Flow Jo Software (Tristar).

### Wound-healing assay

Wound-healing assay was used to assess the effect of digoxin on cell migration. Cells were seeded into 12-well plates (2 × 10^5^ cells per well) and cultured until confluent. The monolayer cells were scratched with a sterile 10 μL pipette tip followed by washing with PBS to remove the floating cells. HCT8 were treated with various concentrations of digoxin for 24 h. HUVECs were treated with supernatant of HCT8 cells pretreated with the indicated concentrations of digoxin for 24 h. Those that migrated to the wounded region were counted. The migration rate was calculated according to the following equation: migration rate (%) = [test counts/control counts (treated with DMSO)] × 100%.

### Transwell-migration assay

Transwell chambers (8-μM pore size; Corning; Corning, NY, USA) were used to confirm the *in vitro* anti-migrative effect of digoxin. Briefly, 1 × 10^6^ cells in the serum-free medium (100 μL) were seeded into the upper chambers and various concentrations of digoxin were added. The lower chamber was supplemented with 650 μL of RPMI medium containing 10% FBS and the same concentration of digoxin as that in the upper compartment. After incubation at 37°C for 24 h, cells were fixed with 90% ethanol, stained with 0.5% eosin, and then observed under the Olympus CKX41 microscope (Olympus; Tokyo, Japan) and photographed.

### Transwell-invasion assay

Transwell-invasion assay was used to examine the effect of digoxin on the invasive ability of HCT8 cells. The upper transwell chambers were pretreated with matrigel (BD Biosciences) to form a filmy barrier. Other procedures and data analysis are the same as in the transwell-migration assay.

### Western-blot analysis

Lysates of cells treated with digoxin or DMSO (control) were prepared. Proteins in the cell lysates were separated by sodium dodecyl sulfate–polyacrylamide gel electrophoresis (SDS–PAGE) and then transferred onto polyvinylidene fluoride membranes (Millipore; Billerica, MA, USA). After being blocked, the membranes were incubated with each primary antibody and then the respective secondary antibody. Signals from the bound antibodies were detected using ChemiDoc XRS+System (Bio-Rad) and quantified using Image J software (NIH) with the respective β-actin signal as background.

### Gelatin-zymography assay

The gelatin-zymography assay was performed to determine the MMP2/9 activity. The HCT8 cells (2 × 10^5^ cells/well) were seeded in six-well plates followed by treatment with digoxin (0.02, 0.05, 0.1 μM) in serum-free RPMI 1640 medium for 24 h. The mediums were collected and concentrated by Amicon Ultra-4 (Millipore). The protein samples were separated by 10% SDS–PAGE containing 0.1% gelatin. Subsequently, the gel was washed with 2.5% Triton X-100 and incubated in reaction buffer at 37°C for 24 h. Then, the gel was stained with Coomassie Brilliant Blue R-250 (Merck; Darmstadt, Germany) in 10% acetic acid and 50% methanol, followed by photography with ChemiDoc XRS^+^ imager (Bio-Rad).

### Enzyme-linked immunosorbent assay

Enzyme-linked immunosorbent assay (ELISA) was performed to investigate the effect of digoxin on VEGF-A secretion by HCT8 cells. Sub-confluent HCT8 cells in a six-well plate were treated with various concentrations of digoxin for 24 h. The culture medium from each well was collected and centrifuged. The supernatants were stored at –20°C to be available for ELISA. A human VEGF-A kit (Jianglai biotech) was used to quantify the VEGF-A in the supernatants. The relative amount in the digoxin-treated cells was expressed as the percentage of the amount in the DMSO-treated control cells.

### Tube-formation assay

The effect of VEGF-A reduction by digoxin on tube formation was determined. Briefly, a pre-chilled 96-well plate was coated with matrigel (BD Biosciences); 100 µL of HUVECs (1 × 10^5^ cells/mL) in medium mixed (1:1) with conditioned medium from digoxin-treated HCT8 cells were seeded in each well of the coated plate. The cells were then incubated for 8 h at 37°C. The capillary-like tubes formed were visualized under an Olympus CKX41 microscope. A representative network of tube structures formed in each well was photographed. For quantification, the total branching points of the tubes were measured using Image J software (NIH). Representative data from three independent experiments were used for analysis.

### Statistical analysis

Data are presented as mean ± standard deviation (SD), representative of at least three independent experiments. One-way ANOVA was used to determine the statistical significance of differences between groups. All statistical analyses were performed using GraphPad Prism 5 software (San Diego, CA, USA) and differences were considered statistically significant when the *P*-value was <0.05.

## Results

### Digoxin inhibited the proliferation of HCT8 and SW620 cells

**Figure 1.**
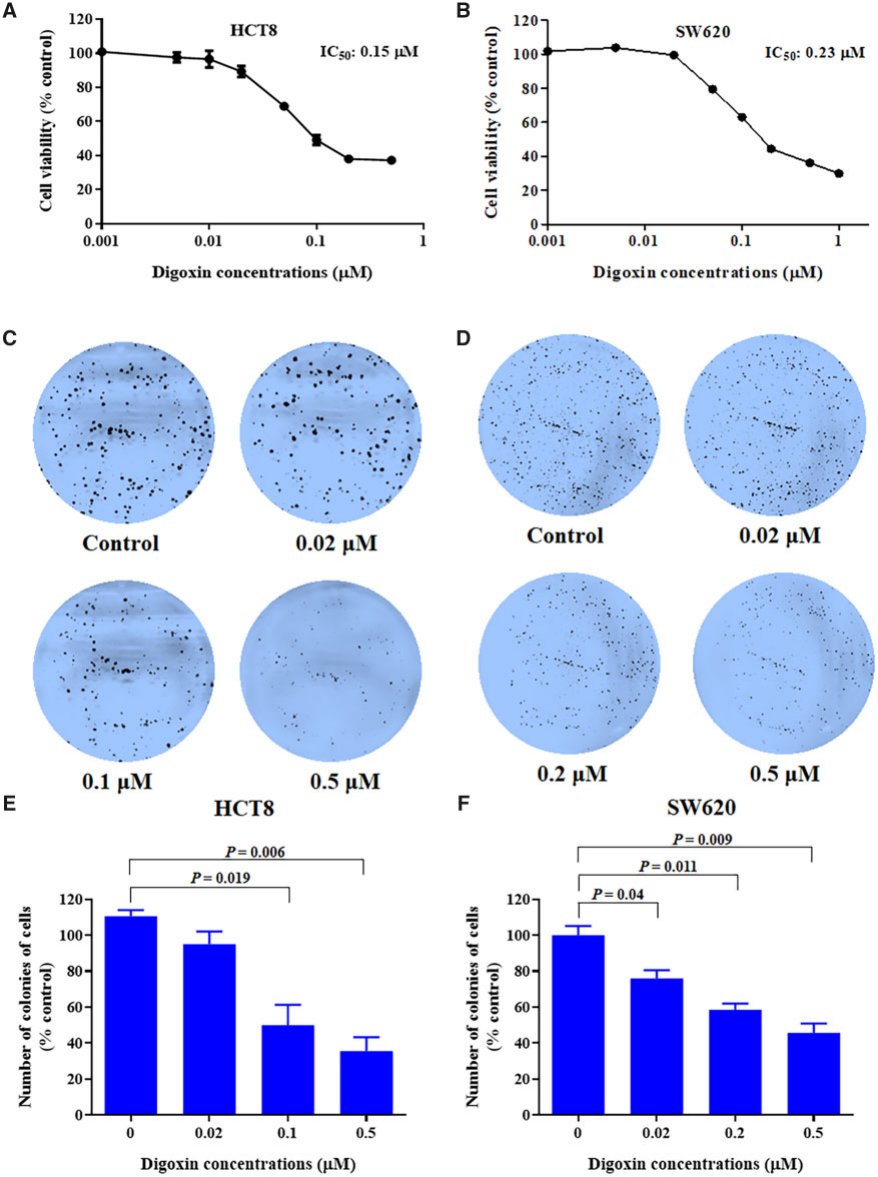
The growth inhibitory effect of digoxin on colorectal-cancer cells. (A) and (B) HCT8 and SW620 cells were treated with various concentrations of digoxin for 24 h, respectively. The cell viability was determined using MTT assay. (C) and (D) Plate colony formation assay was carried out to assess the effect of digoxin on the colony-formation capability of HCT8 and SW620 cells, respectively. After treatment with various concentrations of digoxin, cells were incubated for 12 days and then stained with crystal violet. (E) and (F) The histograms represent the number of colonies of HCT8 and SW620 cells following treatment with digoxin compared to those of control cells. Data are mean ± SD (*n *=* *3), representative of three independent experiments.

The anti-proliferative effect of digoxin on HCT8 and SW620 cells was detected by MTT assay and plate colony formation assay. As shown in Figure 1A and B, the viability of HCT8 and SW620 cells decreased in a dose-dependent manner after exposure to digoxin at a concentration of 0.001–0.5 μM for 24 h, with half maximal inhibitory concentration (IC_50_) values of 0.15 and 0.23 µM for the HCT8 and SW620 cells, respectively. Furthermore, digoxin treatment reduced the number and size of the cell colonies, confirming that digoxin inhibited the proliferation of HCT8 and SW620 cells in a dose-dependent manner, as shown in Figure 1C and F.

### Digoxin induced cell-cycle arrest in HCT8 and SW620 cells

**Figure 2.**
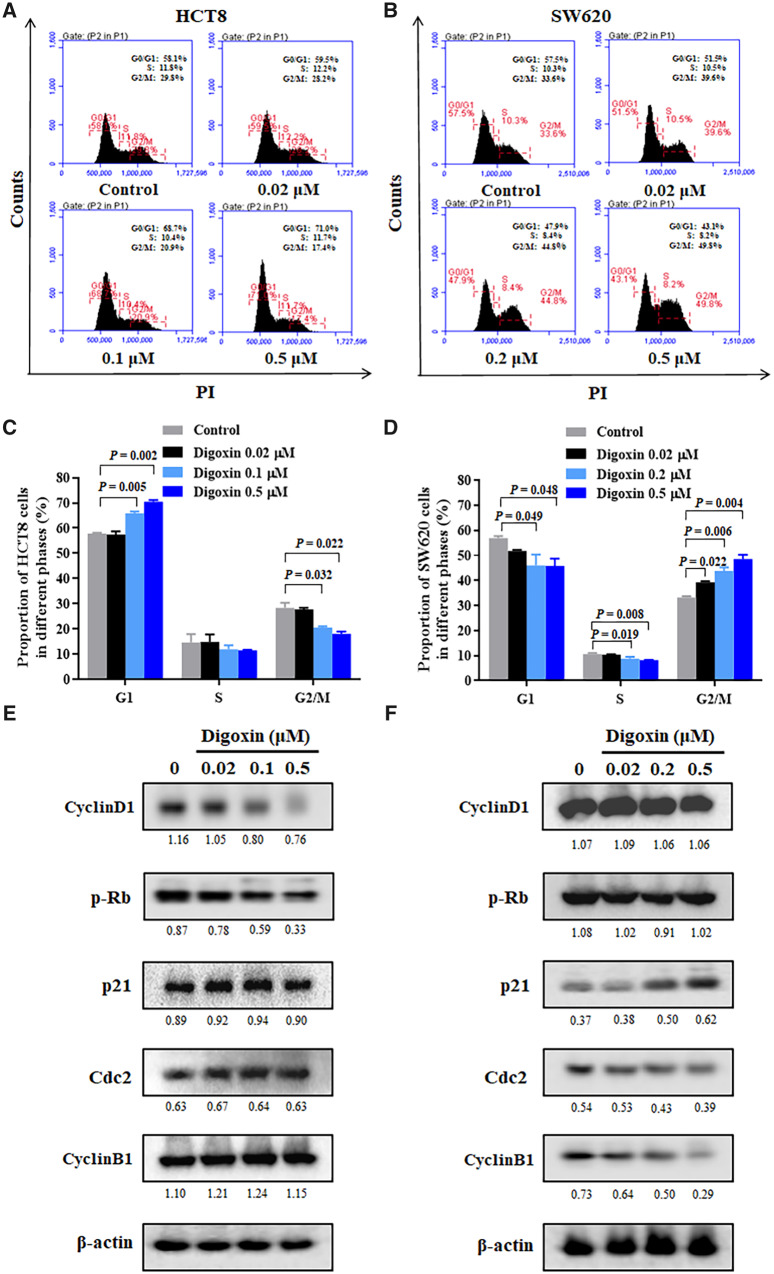
Digoxin induced G1- and G2/M-phase arrest in HCT8 and SW620 cells, respectively. (A) and (B) HCT8 and SW620 cells were incubated with various concentrations of digoxin for 24 h, respectively, and the cell-cycle distribution was determined by flow cytometry. (C) and (D) The percentages of the cell population in G1, S, and G2/M phases were determined. (E) and (F) The levels of CyclinD1, p-Rb, p21, Cdc2, and CyclinB1 were determined by Western blot. Data are presented as mean ± SD, representative of three independent experiments.

We then detected the effect of digoxin on the cell-cycle distribution of HCT8 and SW620 cells by flow cytometry. As shown in Figure 2A and D, after treatment with different concentrations of digoxin for 24 h, cell populations in experimental groups were significantly higher than those in the control group in the G1 phase of HCT8 and the G2/M phase of SW620. To explore the molecular mechanism of digoxin-induced cell-cycle arrest in HCT8 and SW620 cells, we further studied the effect of digoxin on the expression of cell-cycle-related proteins. Figure 2E and F showed that digoxin treatment decreased the expression of CyclinD1 and inhibited the phosphorylation of Rb in HCT8 cells, while increasing the expression of p21 and decreasing the expression of Cdc2 and CyclinB1 in SW620 cells.

### Digoxin did not induce apoptosis in HCT8 and SW620 cells

**Figure 3.**
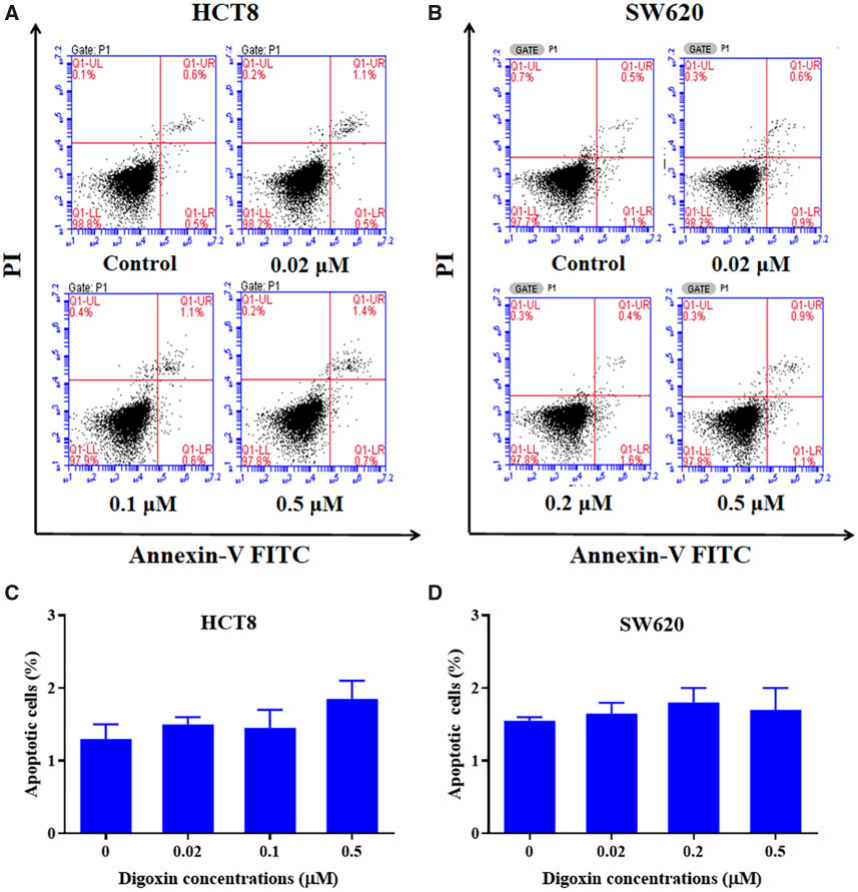
Digoxin did not induce obvious apoptosis in HCT8 and SW620 cells. HCT8 and SW620 cells were treated with indicated concentrations of digoxin for 24 h, stained with Annexin V-FITC and PI (propidium iodide), and then measured by using a flow cytometer.

We further studied whether digoxin can induce apoptosis in HCT8 and SW620 cells. As shown in Figure 3, the number of cells in the treatment groups showed no significant difference with that in the control group. The above results showed that digoxin had no effect of inducing apoptosis in HCT8 and SW620 cells.

### Digoxin inhibited migration and invasion of HCT8 cells

**Figure 4.**
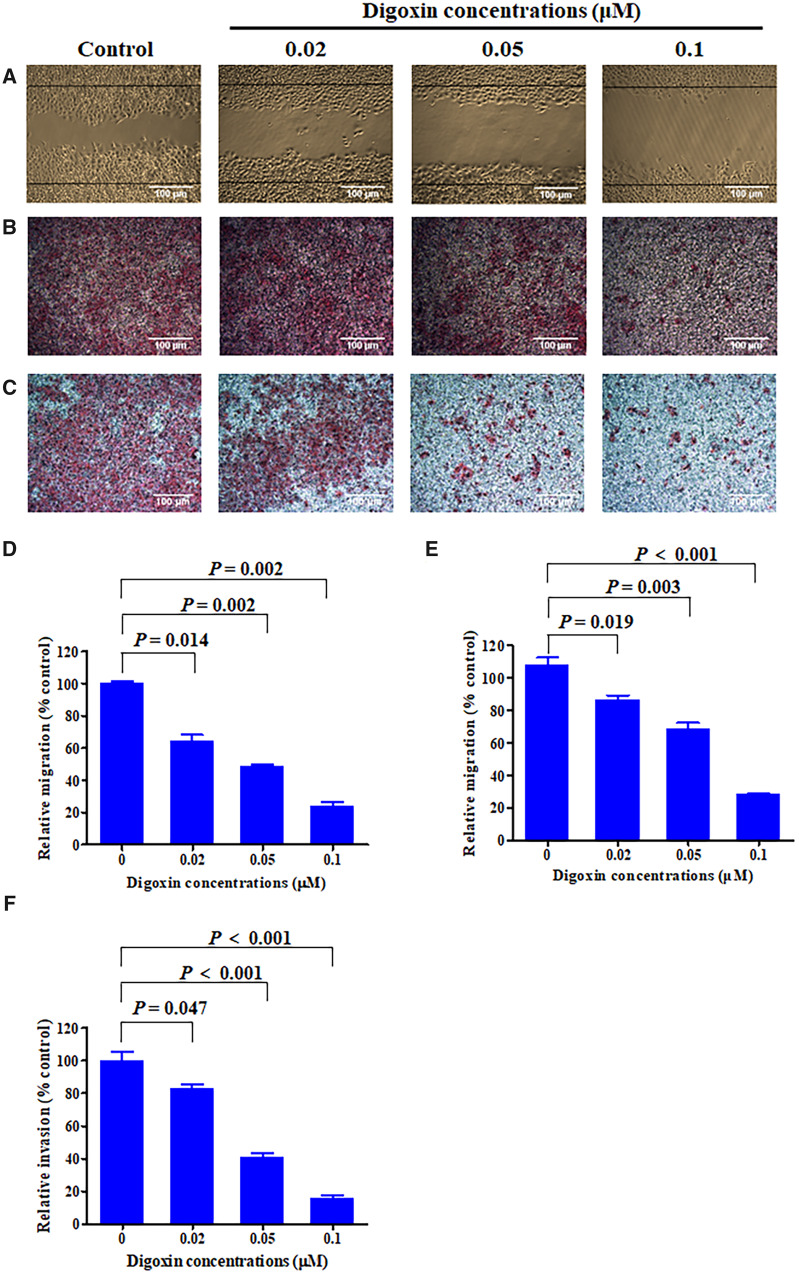
Digoxin inhibited the migration and invasion of colorectal-cancer HCT8 cells. (A) Migration of HCT8 cells with or without digoxin treatment was determined using wound-healing assay. Cells that migrated to the wound area were photographed and counted using inverted microcopy. (B) Cell-migration potency was assessed using transwell-migration assay. After treatment with digoxin for 24 h, the cells that migrated through the transwell-chamber membrane were counted. (C) HCT8 cells were subjected to a matrigel-invasion assay with digoxin treatment as indicated. HCT8 cells that invaded through the matrigel-coated chamber membrane were photographed and counted. (D) Percentages of HCT8 cells that migrated to the wound area following treatment with digoxin relative to those of the control cells. (E) Percentages of cells that migrated after digoxin treatment compared to those of control cells. (F) Percentages of HCT8 cells that invaded through the invasion-chamber membrane after digoxin treatment compared to those of control cells. Data are mean ± SD (*n *=* *3), representative of three independent experiments.

We first tested the anti-migration activity of digoxin against HCT8 cells at a non-cytotoxic concentration. In the wound-healing assay, digoxin inhibited the migration of HCT8 cells after treatment for 24 h (Figure 4A and D). We further examined the effect by using transwell-migration assay. Figure 4B and E show a similar result to the wound-healing experiment, indicating that digoxin inhibited the migration of HCT8 cells in a dose-dependent manner. Then we used transwell-invasion assay to detect the effect of digoxin on the invasive ability of HCT8 cells. After treatment with 0.02, 0.05, and 0.1 μM digoxin, the number of HCT8 cells invading through the membrane was reduced remarkably, indicating that digoxin could block the invasion of HCT8 cells in a dose-dependent manner (Figure 4C and F).

### Digoxin affected the expression of metastasis-related signal proteins and inhibited the expression of HIF1α and the secretion of VEGF-A in HCT8 cells

**Figure 5.**
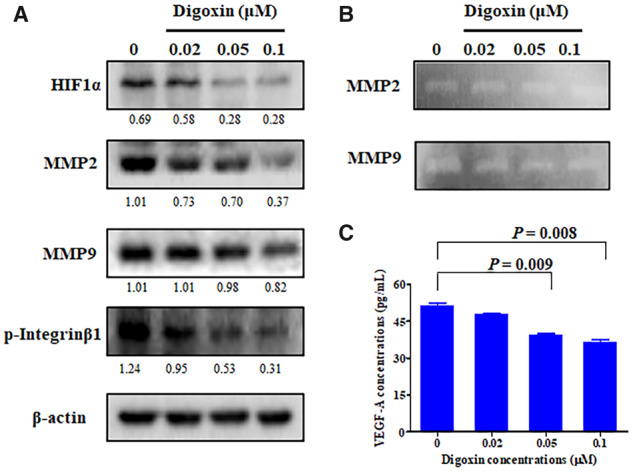
Digoxin exhibited anti-metastatic and anti-angiogenic activities. (A) HCT8 cells were treated with the indicated concentrations of digoxin for 24 h and then the levels of HIF1α, MMP2, MMP9, and p-Integrinβ1 were detected using Western blot. (B) HCT8 cells were treated with the indicated concentrations of digoxin and the MMP2/9 activity was determined using gelatin-zymography assay. (C) VEGF-A (vascular endothelial growth factor A) secretion into the medium of HCT8 cells following treatment with the indicated concentrations of digoxin. Data are mean ± SD (*n *=* *3), representative of three independent experiments.

To explore the potential mechanism of the anti-metastasis of digoxin, we first detected the effect of digoxin on the expression of MMP2, MMP9, and phosphorylated Integrinβ1. As shown in Figure 5A, digoxin decreased the expression of MMP2, MMP9, and phosphorylated Integrinβ1 in HCT8 cells in a dose-dependent manner. Then, we used gelatin zymography to further study the effect of digoxin on the proteolytic activity of MMP2 and MMP9 (Figure 5B) and the results showed that the activities were reduced. These results suggest that the anti-metastatic activity of digoxin may be related to the decrease in expression of MMP2, MMP9, and phosphorylated Integrinβ1.

To investigate whether digoxin can exhibit an anti-angiogenic effect on tumor cells, we detected the expression of HIF1α in HCT8 cells treated with different concentrations of digoxin by Western blot. As shown in Figure 5A, digoxin inhibited the expression of HIF1α in HCT8 cells in a dose-dependent manner. Then, we detected the secretion of VEGF-A protein in the supernatant of HCT8 cells treated with digoxin by ELISA. The result showed that digoxin could inhibit the secretion of VEGF-A protein in a dose-dependent manner (Figure 5C).

### Digoxin reduced angiogenesis of HUVECs

**Figure 6.**
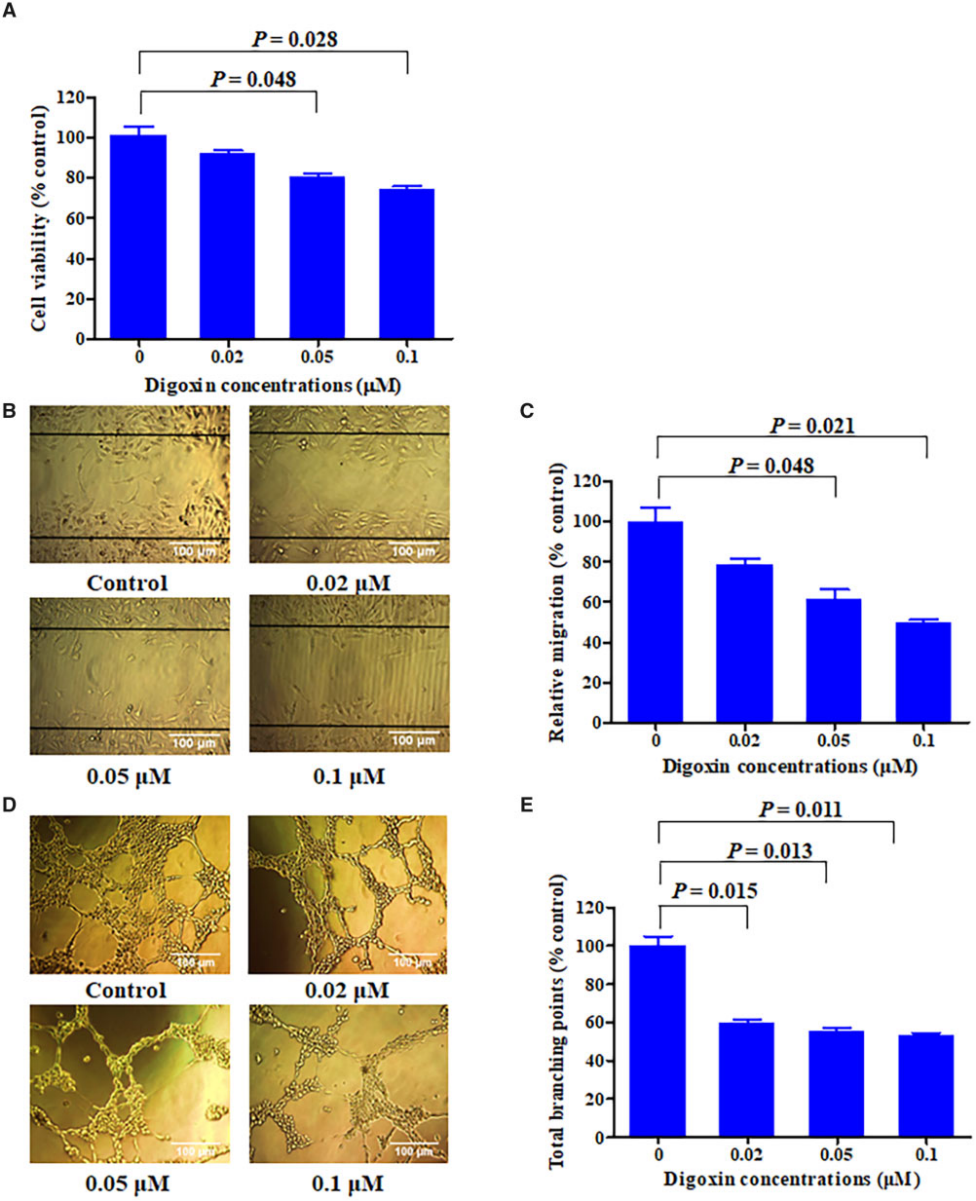
Reduction of VEGF-A by digoxin inhibited the proliferation, migration, and tube formation of HUVECs (human umbilical vein endothelial cells). (A) Proliferation of HUVECs following incubation with the various conditioned media from HCT8 cells after treatment with the indicated concentrations of digoxin. (B) Migration of HUVECs following incubation with the conditioned media collected from HCT8 cells after treatment with the indicated concentrations of digoxin was determined using wound-healing assay. Cells that migrated to the wound area were photographed and counted using inverted microcopy. (C) Migration of HUVECs following incubation with the conditioned media collected from HCT8 cells after treatment with the indicated concentrations of digoxin was determined by transwell-migration assay. (D) Representative images depicting the tube formation by HUVECs following incubation with the conditioned media collected from HCT8 cells after treatment with indicated concentrations of digoxin. (E) Percentage of the total branching points of HUVECs following incubation with the various conditioned media. Data are mean ± SD (*n *=* *3), representative of three independent experiments.

To investigate the effect of digoxin on HCT8 cell-mediated angiogenesis *in vitro*, we first detected the proliferation of HUVECs that were incubated with supernatant from digoxin-treated HCT8 cells for 24 h. As shown in Figure 6A, the supernatant of HCT8 treated with digoxin significantly inhibited the proliferation of HUVECs. Since endothelial-cell migration is very important for angiogenesis, we then examined the effect of digoxin on the migration of HUVECs by the use of wound-healing assay. As a result, digoxin significantly inhibited the migration of HUVECs in a dose-dependent manner (Figure 6B and C).

Subsequently, we constructed a matrigel model to study the effect of digoxin on the tube formation of HUVECs. As indicated in Figure 6D and E, media of the HCT8 cells treated with indicated concentrations of digoxin inhibited tube formation by HUVECs in a dose-dependent manner, demonstrating that digoxin could inhibit the angiogenic effect via reducing VEGF-A secretion by cancer cells.

### Digoxin antagonized multidrug resistance in ABCB1-overexpressing cancer cells

**Figure 7.**
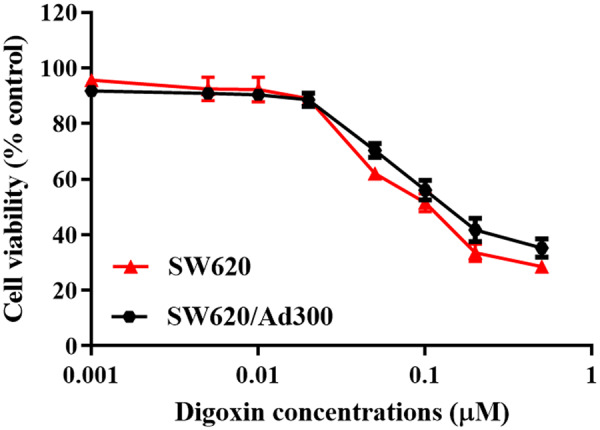
Cell viability of SW620 and SW620/Ad300 cells after treatment with various concentrations of digoxin for 72 h

First, to avoid cytostatic-induced reversal phenomenon, we conducted MTT assays to evaluate the cytostatic effects of digoxin on SW620 and SW620/Ad300 cells. Then, we chose non-cytostatic concentrations for further experiments with 0.01 and 0.02 μM of digoxin (Figure 7). We found that the IC_50_ of digoxin on SW620/Ad300 cells was much higher than that on the parental cells (Table 1). In SW620/Ad300 cells with ABCB1 overexpression, digoxin decreased the IC_50_ of substrate doxorubicin in a concentration-dependent manner (Table 1). In addition, digoxin had no obvious effect on the IC_50_ of cisplatin that is not an ABCB1 substrate in SW620 and SW620/Ad300 cells (Table 1). The above results showed that digoxin can specifically enhance the sensitivity of ABCB1-overexpressing cancer-cell SW620/Ad300 to doxorubicin, suggesting digoxin reversed multidrug resistance in the cells.

**Table 1.** Effect of digoxin on chemotherapeutic agents in ATP-binding cassette (ABC) transporter-expressing cells *in vitro*

	IC_50_ ± SD (μM)^a^ (RF)^b^	
Treatment	SW620	SW620/Ad300
Doxorubicin	0.160 ± 0.092 (1.00)	23.275 ± 2.592 (1.00)
+Digoxin (0.01 μM)	0.116 ± 0.033 (1.38)	11.569 ± 2.265 (2.01)
+Digoxin (0.02 μM)	0.125 ± 0.063 (1.28)	6.703 ± 1.184 (3.47)*
+Verapamil (10 μM)	0.111 ± 0.013 (1.45)	0.397 ± 0.097 (58.71)**
Cisplatin	3.636 ± 0.424 (1.00)	9.436 ± 0.953 (1.00)
+Digoxin (0.01 μM)	3.081 ± 0.813 (1.18)	9.362 ± 1.949 (1.01)
+Digoxin (0.02 μM)	3.033 ± 1.254 (1.20)	7.373 ± 0.501 (1.28)

Verapamil was employed as a ABCB1-positive control inhibitor.

^a^ IC_50_ values were determined by MTT assay as described in the ‘Materials and methods’ section and were obtained from three independent experiments in triplicate.

^b^ Resistance fold (RF) was calculated by dividing the IC_50_ values of parental cells or resistant cells by the IC_50_ of parental cells or resistant cells in the absence of digoxin or a positive control inhibitor. *P < 0.05; **P < 0.01, compared with doxorubicin-only treatment group.

## Discussion

In this paper, we found that digoxin potently inhibited proliferation and induced G1-phase and G2/M-phase arrest for HCT8 and SW620 cells, respectively. But no obvious apoptosis was induced in these cells. In addition, digoxin exhibited anti-metastasis activities on HCT8 cells by inhibiting the migration and invasion of HCT8 cells. On the other hand, digoxin indicated an anti-angiogenic effect by inhibiting the proliferation, migration, and tube formation of HUVECs. Finally, digoxin specifically reversed ABCB1-mediated multidrug resistance in SW620/Ad300 cells.

Digoxin and other cardiac glycosides have been assessed in clinical trials against various types of tumors [14, 15, 17]. Although early study reported that the use of digoxin might be associated with increased CRC risk [18], recent research has proved that digoxin was safe for CRC patients [16].

Digoxin has been reported to inhibit the proliferation of CRC cells HT29 and HCT116, alone or in combination with oxaliplatin [19]. In that report, the molecular mechanism was not elucidated, but was unrelated to the inhibition of Na^+^/K^+^-ATPase [19]. In this paper, we found that digoxin exerted an anti-proliferative effect on HCT8 and SW620 cells independently of apoptosis. Interestingly, digoxin induced G1 arrest in HCT8 cells by decreasing the expression of CyclinD1 and phosphorylated Rb, but induced G2/M arrest in SW620 cells by decreasing the expression of Cdc2 and CyclinB1, and increasing the expression of p21.

On the other hand, digoxin showed anti-migration and anti-invasion activity at lower concentrations than cytotoxic IC_50_ of HCT8 (0.15 μM). Digoxin reduced the expression of MMP2, MMP9, and phosphorylated Integrinβ1 in HCT8 cells. The proteolytic activity of MMPs was also inhibited by digoxin treatment. It is known that MMP2 and MMP9 can degrade gelatin and Type IV collagen, and break the barriers so that cancer cells can traverse to metastasis [20]. And Integrinβ1 could switch the tumor from dormancy to metastatic growth [21]. Therefore, regulation of the above molecules might contribute to the anti-metastasis effect of digoxin on colorectal cancer.

We also found that digoxin inhibited HIF1α expression and VEGF-A secretion in HCT8 cells, since HIF1α was known to promote the expression of VEGF-A, which is an angiogenesis factor [22]. These results suggested that digoxin had a potential anti-angiogenic activity via blocking the HIF1α expression and VEGF-A secretion in HCT8 cells.

Tumor angiogenesis is a complex process, including endothelial-cell proliferation, migration, and lumen formation. As the first anti-angiogenic drug, bevacizumab was approved by the US Food and Drug Administration for the treatment of CRC. Our result indicated that digoxin also exerted anti-angiogenesis via the blockade of VEGF-A secretion in HCT8 cells, via inhibiting the proliferation and migration of vascular endothelial cells.

**Figure 8.**
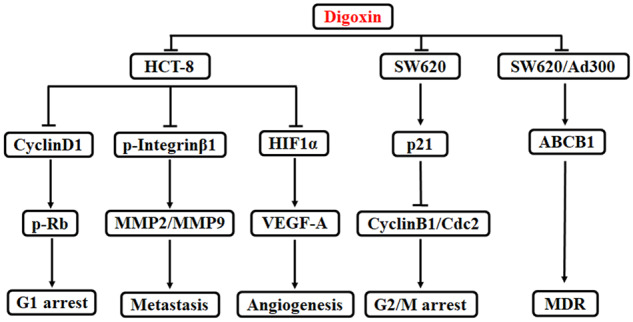
Schematic illustration of the mechanism of digoxin on HCT8 and SW620 cells

Above all, digoxin has the potential to inhibit proliferation, migration, invasion, and angiogenesis, and to reverse multidrug resistance in CRC cells. The mechanism of digoxin on HCT8 and SW620 cells has been illustrated in Figure 8. Therefore, digoxin is expected to become a promising drug candidate for the therapy of CRC.

## Authors’ contributions

Z.Z., W.W., Z.S.C., and D.X.K. conceived and designed the project. Y.Q.H., Y.Y.W., and X.C.W. performed the experiments and collected the data. Y.L. and C.Z.Z. analysed and interpreted the data. Z.Z. drafted the manuscript. W.W. and D.X.K. edited the manuscript. All authors read and approved the final manuscript.

## Funding

This work was supported by the Science & Technology Development Fund of Tianjin Education Commission for Higher Education [2017KJ230].
